# A new semi-supervised learning model combined with Cox and SP-AFT models in cancer survival analysis

**DOI:** 10.1038/s41598-017-13133-5

**Published:** 2017-10-12

**Authors:** Hua Chai, Zi-na Li, De-yu Meng, Liang-yong Xia, Yong Liang

**Affiliations:** 1 Faculty of Information Technology & State Key Laboratory of Quality Research in Chinese Medicines, Macau University of Science and Technology, Avenida Wai Long,Taipa, Macau, 999078 China; 2 Institute for Information and System Sciences and Ministry of Education Key Lab of Intelligent Networks and Network Security, Xi’an Jiaotong University, Xi’an Shaan’xi, 710049 China

## Abstract

Gene selection is an attractive and important task in cancer survival analysis. Most existing supervised learning methods can only use the labeled biological data, while the censored data (weakly labeled data) far more than the labeled data are ignored in model building. Trying to utilize such information in the censored data, a semi-supervised learning framework (Cox-AFT model) combined with Cox proportional hazard (Cox) and accelerated failure time (AFT) model was used in cancer research, which has better performance than the single Cox or AFT model. This method, however, is easily affected by noise. To alleviate this problem, in this paper we combine the Cox-AFT model with self-paced learning (SPL) method to more effectively employ the information in the censored data in a self-learning way. SPL is a kind of reliable and stable learning mechanism, which is recently proposed for simulating the human learning process to help the AFT model automatically identify and include samples of high confidence into training, minimizing interference from high noise. Utilizing the SPL method produces two direct advantages: (1) The utilization of censored data is further promoted; (2) the noise delivered to the model is greatly decreased. The experimental results demonstrate the effectiveness of the proposed model compared to the traditional Cox-AFT model.

## Introduction

Disease related gene selection has great potential in outcome prediction for cancer research. The identified gene and constructed disease related network based on these genes^1^ has been widely used in cancer prediction^2^, classification^3^, treatment^4^ and gene-targeting drug development^5^. How to accurately select the pathogenic gene is an attractive and important task in cancer research. Various methods have been used to solve this problem, including Cox proportional hazards model (Cox)^6^, accelerated failure time model (AFT)^7^, cancer hallmark approach^8^ and construct network motifs as cancer biomarkers^9^.

The high dimension and low sample size of biological data greatly increase the difficulty of cancer survival analysis. It is statistically challenging because the number of genes is far larger than that of the labeled samples. To solve this problem, many supervised learning methods have been designed by using different kinds of regularization methods, such as elastic net^10^, L_1_ regularization^11^, L_1/2_ regularization^12^, minimax concave penalty (MCP)^13^, smoothly clipped absolute deviation (SCAD)^14^ and so on. Meanwhile we cannot ignore the censored data in the biological dataset. Censored data means that the observed time is not the true survival time, and for such data we only know the fact that the actual survival time is longer than the observed time. Nevertheless, many researchers have pointed out the information underlying the censored data are very helpful for model building^15^. Hence some semi-supervised learning methods such as^16,17^ were proposed to utilize the censored data and have achieved better results than the conventional supervised learning methods. While those methods are mainly based on the logistic model or SVM model. In ^18,^ a novel semi-supervised learning framework integrating the Cox model and AFT model was proposed to solve the following two dilemmas:

##  Few available data versus high dimensional covariates dilemma

The Cox model is one of the most widely used methods in cancer analysis which can assess patients’ survival risk and classify the patients into ‘high risk’ and ‘low risk’ groups using the gene expression profile. However, the lack of enough information in the labeled dataset tends to conduct the issue of the inaccuracy of prediction. Trying to solve this dilemma, the AFT model is employed to estimate the true survival time for the censored data, and therefore more disease information in the censored data can be delivered to the Cox model, which can help Cox model to produce better predictions.

##  Similar phenotype disease data versus different genotype cancer dilemma

Recent research pointed out that similar phenotype cancers may be completely different diseases on the molecular genotype level^19,20^, and hence the AFT model cannot use some cancer data which have the same phenotype directly but with different molecular genotypes directly. The Cox-AFT model alleviated this dilemma by using the Cox model to classify the cancer data firstly because the cancers with different molecular genotype levels may lead to the different risks of the patients.

In the Cox-AFT model, the Cox model was used to classify the similar phenotype disease data into ‘low risk’ and ‘high risk’ subgroups, and these subgroups will be sent into the specific AFT model to get approximate estimate of survival time for the censored data. At last, these pseudo labeled censored data will be fed into the Cox model as labeled data.

Though effective to some extent, the Cox-AFT model suffers from the robust issues caused by heavy noise and even outliers. We found that many censored data always violate the constraint that the estimated survival time is supposed to be longer than the censored time. Therefore these falsely labeled samples are dismissed in this model, which restricts the full exploitation of the censored data. Furthermore, the samples satisfying the constraint may not be estimated correctly in the stage of the AFT model. Fed with such data with label noise, the Cox model may be evidently degenerated and its performance may be more or less harmed to the next training cycle.

The reliability and stability of the Cox-AFT model relies heavily on the accuracy of the AFT model. However, the single AFT model always encounters the robust issue in semi-supervised learning scenarios. In the first few iterations of the AFT model, censored samples have high chance to be wrongly labeled due to the inaccurate model parameters. Worse still, the AFT model utilizes all the labeled censored data to conduced model learning, and as a result the noisy information remains in the following iterations. Therefore the selection of samples values a lot in the training of the AFT model.

To solve the issues mentioned above, we introduce a robust learning mechanism called self-paced learning (SPL). The self-paced learning^21^ was proposed based on the core idea of the curriculum learning (CL)^22^. Curriculum learning (CL) simulates the learning process of human beings and tend to learn easy samples first and then gradually include more complex samples into training process. The challenge in CL is the requirements of the prior knowledge about the sample easiness order. Compared to CL, SPL can identify the easy and hard samples adaptively according to what the model has already learned and gradually add harder samples into training. The SPL method has been used successfully in multiple machine learning tasks^23–25^. Moreover,^26^  has proved the robust insight of SPL regime, by proving the equivalence between the optimization of SPL objective function and the majority minimization of a non-convex penalty. Hence SPL is a powerful robust learning regime to help us estimate the patients’ survival time more accurately.

In this paper we introduce the SPL regime in the Cox-AFT model (Cox-SP-AFT), largely improving the model capacity in the presence of heavy noises and outliers. SPL is embedded into the AFT model and takes effect by automatically selecting samples following the “easy” to “hard” mode in the training process, which means learning samples of high confidence first and gradually considering more complex ones. This learning mechanism leads to more accurate estimation for the censored samples compared to that without SPL and brings many benefits. A comparison experiment between Cox-AFT models with and without considering SPL is shown in the Experiment section. It is verified that the Cox-SP-AFT model can select more correct disease-related genes, estimate the patients’ survival time more accurately and employ more censored data, validating the superiority of our proposed semi-supervised learning model with SPL.

## Method

Suppose that the dataset includes *l* samples consisting of complete dataset and censored dataset to study the correlations between the gene expression profile *X* and according survival time Y. ${({t}_{i},{\delta }_{i},{x}_{i})}_{i=1}^{l}$ represents an individual patient’s sample, where *t*_*i*_ is the observed time, and ${x}_{i}=({x}_{i1},{x}_{i2},\ldots {x}_{ip})$ is the gene expression profile. If ${\delta }_{i}=0,$ it represents *t*_*i*_ is the censored time; If ${\delta }_{i}=1,$ it means *y*_*i*_ is the labeled time.

### Cox proportional hazard model

The Cox proportional hazard model is used to classify the patients into two groups of the ‘low risk’ and ‘high risk’, and the baseline hazard function can be expressed as:

1 $$h(t|\beta )={h}_{0}(t)\exp ({\beta }_{i1}{x}_{i1}+{\beta }_{i2}{x}_{i2}+\ldots +{\beta }_{ip}{x}_{ip})={h}_{0}(t)exp({\beta }^{T}x).$$

Minimizing the Cox’s partial log likelihood function:

2 $$l(\beta )=\sum _{i=1}^{n}\,{\delta }_{i}\{{x}_{i}^{T}\beta -log[\sum _{j\in {R}_{i}}\,\exp ({x}_{j}^{T}\beta )]\},$$

where the ordered risk set at time *t*_*i*_ can be denoted by ${R}_{r}=\{j\in 1,\ldots ,n:{t}_{j} > {t}_{i}\}.$

In fact, some correlation coefficients *β*_*i*_ of the *i* ^th^ gene may be zero in the true model, which means that not the whole covariates have effect to the prediction. Therefore the model should be able to identify the nonzero coefficients in the gene expression profile; a regularization part was added to solve this problem. So the penalized Cox model with penalty function can be expressed as:

3 $${\beta }^{\ast }=\arg \,\mathop{min}\limits_{\beta }\{l(\beta )+\lambda \sum _{j=1}^{p}P({\beta }_{j})\},$$

where λ is a tuning parameter and *P*(β) is the regularization term.

In recent years, methods with different regularization terms such as elastic net, L_1_, MCP and L_1/2_ have been used in cancer survival analysis. In our semi-supervised learning model, we use the MCP regularization. This combination has good performance in sparsity and data-fitting ability. The derivative of the MCP can be expressed as:

4 $${P}_{\gamma }(\beta ;\lambda )=\{\begin{array}{c}\lambda |\beta |-\frac{{\beta }^{2}}{2\gamma },\,if\,|\beta |\le \gamma \lambda ,\\ \frac{1}{2}\gamma {(\lambda )}^{2},\,if\,|\beta | > \gamma \lambda .\end{array}\,$$

### Accelerated Failure Time (AFT) model

The AFT model is a log-linear regression model which can be used to predict the patients’ survival time:

5 $${\rm{l}}{\rm{o}}{\rm{g}}\,{t}_{i}={\beta }_{1}{x}_{{\rm{i}}1}+{\beta }_{2}{x}_{i2}+\ldots +{\beta }_{p}{x}_{ip}+{\varepsilon }_{i}.$$

In the AFT model of our model, censored data are initially labeled using the Kaplan-Meier weight estimator because it’s simple and fast. Trying to get more accurate results in a robust way, the SPL regime is integrated in the AFT model.

### Self-Paced Learning

Curriculum Learning was first proposed in^16,17^, which follows the learning principle of humans. Afterwards,^16^ formulates the key principle of CL as a concise optimization model through introducing a regularization term. The SPL objective function includes a weighted loss term on all samples and a general self-paced regularization can be expressed as:

6 $$\mathop{min}\limits_{\omega ,V\in {[0,1]}^{n}}E(\omega ,V;\lambda )=\sum _{i=1}^{n}({v}_{i}L({y}_{i},g({x}_{i},\omega ))+f({v}_{i},\lambda )),$$

$D={\{({x}_{i},{y}_{i})\}}_{i=1}^{n}$ denotes the training data set, where *x*_*i*_ is the *i* ^*th*^ training sample and *y*_*i*_ is the according label. $L({y}_{i},g({x}_{i},\omega ))$ represents the loss function of *x*_*i*_. $g({x}_{i},\omega )$ is the decision function, and ***ω*** is the model parameter inside. $V=({v}_{1},\mathrm{...},{v}_{n})$ is a weight vector of all samples. *λ* is the age parameter for controlling the learning pace, and $f(v,\lambda )$ is the self-paced regularization term imposed on the sample weight. By optimizing the weight vector *V* with gradually increasing age parameter, more samples can be automatically selected into the training process from easy to complex in a purely self-paced way. There are several variants to the original hard regularization function $f(v,\lambda )=-\lambda v$, such as the linear and mixture SP regularization in^21^.

### SP-AFT Model

The SP-AFT model updates the original AFT model through additionally embedding the SPL regime, and inherits the priorities of SPL method, such as better robustness and higher accuracy. The specific objective function of SP-AFT model is given by adding weights to the censored data as well as a self-paced regularization term:

7 $$\mathop{min}\limits_{\begin{array}{c}\beta ,{y}_{j},{v}_{j}\in \{0,1\}\\ j=n+1,\cdots ,n+m\end{array}}\sum _{i=1}^{n}l({t}_{i},{x}_{i}^{T}\beta )+\sum _{j=n+1}^{n+m}({v}_{j}l({y}_{j},{x}_{j}^{T}\beta )+f({v}_{j},\alpha ))+\lambda P(\beta ),$$

where *m*, *n* are the numbers of labeled samples and censored samples, respectively. ${\{{x}_{i},{t}_{i}\}}_{i=1}^{n}$ is the labeled dataset with ${\delta }_{i}=1$, and ${\{{x}_{j},{t}_{j}\}}_{j=n+1}^{n+m}$ is the censored dataset with ${\delta }_{i}=0$. $\beta ,\,{{\rm{y}}}_{j},\,{v}_{j},\,{l}_{j}(j=n+1,\mathrm{...},n+m)$ are the model parameter, label variable, the weight term and the loss for the censored sample $({x}_{j},{t}_{j})$. $f({v}_{j},\alpha )=-{\rm{\alpha }}{v}_{j}\,\,$denotes the self-paced regularization term imposed on the weight term *v*_*j*_ as well as the age parameter *α*. The age parameter controls the learning pace and the larger value will allow more complex samples into training. $P(\beta )$ represents the MCP regularization term on *β*.

Alternate Optimization Search(AOS) algorithm is adopted to optimize SP-AFT model. The detailed optimization procedure is presented below:

#### Initialize

Some optimization variables and parameters are preset in this step. For the censored data set, the survival time of each sample is estimated with the Kaplan-Meier method. ${V}^{0}=({v}_{n+1},\mathrm{...},{v}_{n+m})$ is an all-one vector of ${R}^{m}$. *λ* is set to a small value to include several samples into training in the first round.

#### Update *β*^(*t*)^

*β* will be updated by the AFT model with the MCP regularization utilizing the complete data and censored data with non-zero weight. In this implementation, loss function is adopted as follows:

8 $$l({y}_{{\rm{j}}},{x}_{j}^{T}\beta )=\{\begin{array}{c}\begin{array}{cc}+{\rm{\infty }} & \begin{array}{cc}if & {t}_{j} > {x}_{j}^{T}\beta \end{array}\end{array},\\ \begin{array}{cc}{({y}_{j}-{x}_{j}^{T}\beta )}^{2} & otherwise.\end{array}\end{array}$$

This loss function is derived from the constraint that the survival time must be no less than the censor time. Therefore, if the estimated survival time of a sample is less than the censor time, this sample must be falsely labeled and its loss value is positive infinity. However, if a censored sample obeys the censor condition, its loss function is square loss. Therefore, Formula (7) degenerates to the following AFT model:

9 $${\beta }^{(t)}=\mathop{\arg \,min}\limits_{\beta }\sum _{{\rm{j}}\in I}l({y}_{j}^{(t-1)},{x}_{j}^{T}\beta )+\gamma P(\beta ),$$

Where *I*  denotes the sample set of complete data and censored data with non-zero weight ${v}_{j}^{(t-1)}$. We employ the minimax penalty here and the off-the-shelf methods to solve (9).

#### Update ${v}_{j}^{(t)}$

The physical meaning of this step is to select confident samples from the censored dataset according *v*_*j*_. With this step of selecting high-confidence samples, the robustness of SP-AFT can be largely improved compared to that of the AFT model. Calculate the derivative with respect to ${v}_{j}$ of (7):

10 $$\frac{{\rm{\partial }}E}{{\rm{\partial }}{v}_{j}}=l({y}_{j}^{(t-1)},{x}_{j}^{T}{\beta }^{(t)})-\alpha .$$

Through such simple calculation, we can get the closed-form updating equation for ${v}_{j}$:

11 $${v}_{j}^{(t)}=\{\begin{array}{cc}1 & {l}_{j}^{(t)}\le \alpha ,\\ 0 & otherwise.\end{array}$$

The samples with losses smaller than age parameter *α* will be seen as ‘easy’ ones and assigned as ${v}_{j}=1$; Otherwise will be signed as ${v}_{j}=0$.

At the first start, the weight values of the censored data are all set to 1, and we suppose that they are confident samples in the first iteration because the Kaplan-Meier estimate is a good but primary approximation to the real survival time. In the following iterations, confident samples are selected according to the loss value. A sample with loss value no more than the age parameter *λ* will be picked.

#### Update ${y}_{j}^{(t)}$

In this step, we update the estimated survival time for the censored data with the learned parameter *β* ^(*t*)^ as well as the weight:

12 $${y}_{j}^{(t)}=\{\begin{array}{c}\begin{array}{cc}{x}_{j}^{T}\beta  & {v}_{j}^{(t)}=1,\end{array}\\ \begin{array}{cc}{y}_{j}^{(t-1)} & {v}_{j}^{(t)}=0.\end{array}\end{array}$$

This updating formula indicates that if ${v}_{j}^{(t)}=1$, the sample ${x}_{j}$ will be assigned the newly estimated survival time. Otherwise, the estimated value will remain unchanged. Once the censored samples are pseudo estimated, the age parameter $\lambda$ is enlarged to include more censored samples with larger losses into training. The iteration will stop until convergence.

**Figure:**
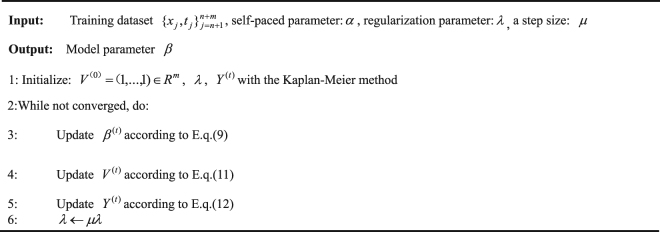
The Algorithm of SP- AFT Model.

### Cox-SP-AFT model

**Figure 1:**
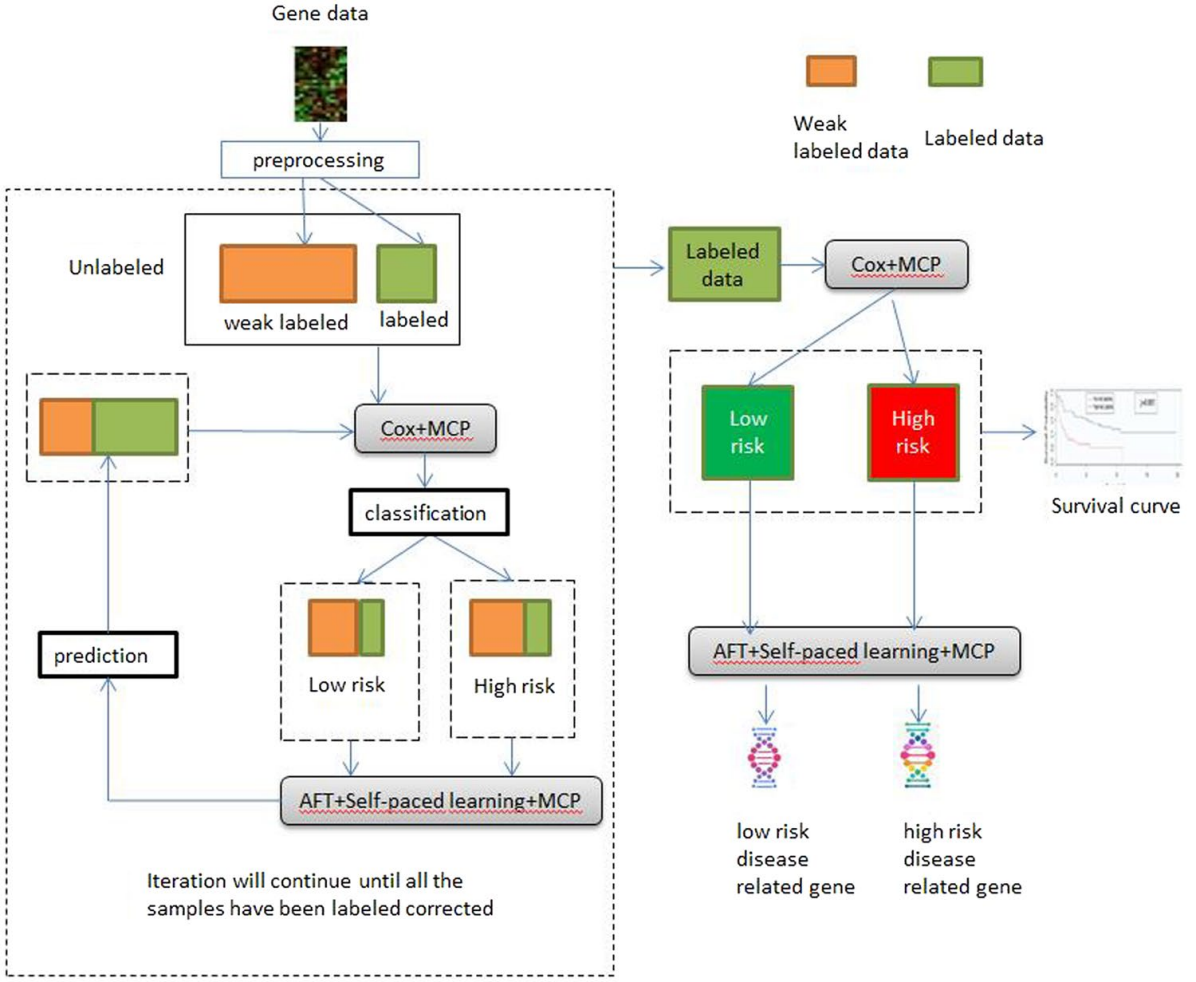
The workflow of our proposed semi-supervised learning model with SPL.

The work flow of our proposed semi-supervised learning model is shown in Fig. 1. In a training round of Cox-SP-AFT model, the training samples are firstly put into the Cox model penalized with MCP regularization, and the constructed model will classify the whole dataset into ‘high-risk’ and ‘low-risk’ groups. Then the two groups will be sent into their according SP-AFT models, respectively. In the SP-AFT model, the survival time of the censored data will be estimated. However, some estimated time of censored samples were less than the censored times. It is obvious that these censored samples were wrongly labeled. Thanks to the mechanism of SPL method, these samples with large losses will be automatically assigned zero weights and take no effect in the next iteration. At the terminal iteration of SP-AFT, reliable labeled samples (with non-zero weight) will be added to the labeled dataset thus updating the training set of the next Cox-SP-AFT round. The algorithm of our proposed Cox-SP-AFT model is outlined below:

**Figure:**
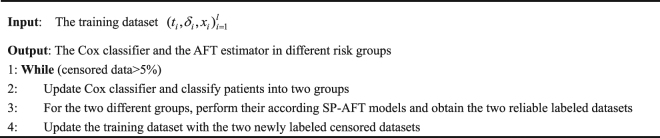
The Algorithm of Cox-SP-AFT Model.

## Results

We designed the simulation scheme as in^27^. The simulation data were generated as following:

Step 1: We set the dimension of the genes *p* = 2000, in which 20 corresponding coefficients of the related genes were nonzero, and the coefficients of the remaining 1980 genes were zero. The censored rate *k* was set 0.5; the correlation coefficients *c* was set 0.3. The number size *n* of the whole dataset was 250.

Step 2: We generate the ${{\rm{\gamma }}}_{i0},{\gamma }_{i1},\ldots ,{\gamma }_{ip}$ (i = 1, …, n) independently from standard normal distribution, the X was set ${X}_{ij}={\gamma }_{ij}\sqrt{1-c}+{\gamma }_{i0}\sqrt{c}$.

Step 3: The survival time was computed as: ${y}_{i}=\frac{1}{\alpha }\,\mathrm{log}(1-\frac{\alpha \ast \mathrm{log}(U)}{\omega \ast \exp (\beta X)})$, where *U* is the uniformly distributed variable, $\alpha$ is the shape parameter and the $\omega$ is the scale parameter.

Step 4: The censored time point was decided in random selection, and the censored time ${y^{\prime} }_{i}$ was computed as ${y^{\prime} }_{i}={rand}(1)\,\ast \,{y}_{i}$, we recorded the (${y}_{i},{y^{\prime} }_{i},{X}_{i},{\delta }_{i}$), where the ${y}_{i}$ is the true survival time, ${y^{\prime} }_{i}$ is the observed time, ${X}_{i}$ is the gene expression profile and ${\delta }_{i}$ represent the data is censored or not.

We generated 10 datasets through setting different *β* values of random selected genes, 200 random selected samples in each dataset were used as the training data and the remaining 50 samples were used as the test data each time, in this paper we compared five methods including three supervised learning and two semi-supervised learning models, the supervised learning methods were penalized by elastic net, lasso and MCP respectively. The difference between the two semi-supervised learning models is they contain the self-paced learning or not. Different methods in each dataset were evaluated 100 times and the average results were shown in below.

Table 1 is the number of the selected correct genes obtained by five different Cox methods, three supervised learning methods: the elastic net penalized Cox model (Cox-EN), the lasso penalized Cox model (Cox-lasso) and the Cox mode with MCP (Cox-MCP), the other two methods are Cox models in semi-supervised learning models with or without self-paced learning (Semi-Cox or SP-Semi-Cox). The last row shows the average values of the results obtained by different methods. We can find the number of selected correct genes obtained by Cox-lasso and Cox-MCP are nearly the same; the Cox-EN selected more correct genes than the Cox-lasso and Cox-MCP. However the performance of the semi-Cox without SPL is better than Cox-EN, and it is obviously that the SP-Semi-Cox model selected most correct genes.

**Table 1:** The number of the selected correct genes obtained by different methods.

Dataset	Cox-EN	Cox-lasso	Cox-MCP	Semi-Cox	SP-Semi-Cox
1	9.86	6.69	6.74	12.18	12.66
2	11.32	7.58	7.51	13.48	14.27
3	16.78	12.31	12.04	19.43	19.83
4	10.21	6.70	6.75	11.57	11.91
5	13.72	9.96	9.88	15.22	16.35
6	9.25	6.18	6.26	13.14	14.29
7	12.39	8.32	8.17	14.60	16.33
8	13.02	8.46	8.39	15.32	16.82
9	11.40	7.42	7.54	14.71	15.56
10	15.39	10.26	10.21	18.31	19.29
Average	12.33	8.39	8.35	14.80	15.73

The numbers of the total selected genes obtained by different methods were shown in Table 2, the Cox-EN selected most genes, it means there may be many genes unrelated to disease. The results obtained by Cox-lasso and Semi-Cox are nearly the same, the SP-Semi-Cox selected less genes compared to the Semi-Cox without SPL but more than the Cox-MCP, and the supervised learning method Cox-MCP selected least genes.

**Table 2:** The number of the total selected genes obtained by different methods.

Dataset	Cox-EN	Cox-lasso	Cox-MCP	Semi-Cox	SP-Semi-Cox
1	87.46	52.73	34.21	56.73	48.35
2	72.68	40.32	25.56	40.30	32.28
3	135.22	96.35	60.41	88.61	80.74
4	58.36	34.07	20.93	35.50	30.25
5	121.79	86.43	52.66	73.91	68.17
6	70.26	36.59	21.78	38.25	33.18
7	80.47	48.95	33.52	54.32	49.83
8	66.58	40.09	23.36	41.87	35.62
9	98.39	62.71	44.63	60.38	56.35
10	50.41	31.24	17.36	30.47	26.44
Average	84.17	52.95	30.44	52.03	46.12

**Figure 2:**
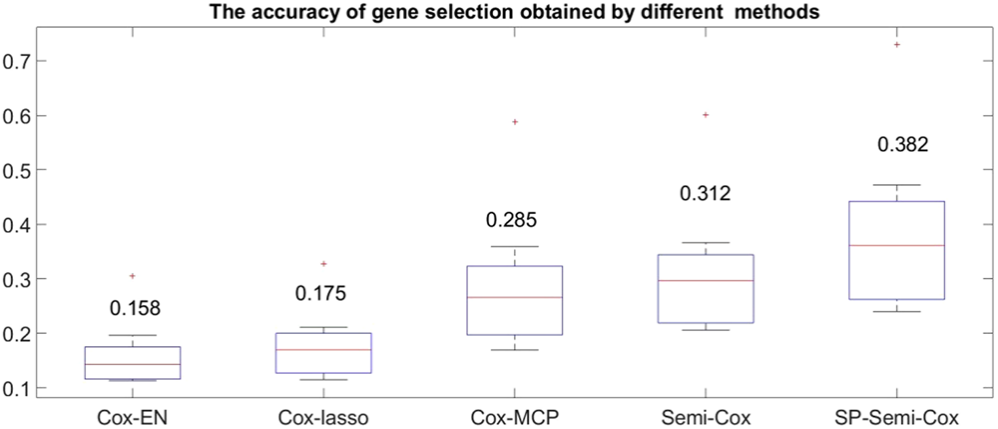
The gene selection accuracy obtained by different methods.

The accuracy of correct gene selection obtained by different methods were shown in Fig. 2, it is obviously that the accuracy obtained by the SP-Semi-Cox is highest in the five methods. The accuracy of Semi-Cox is higher than other three supervised learning methods, and the accuracy of Cox-EN is lowest because it selected many unrelated genes. Compared the performances we can say though the Cox-MCP selected fewer genes than SP-Semi-Cox, but it cannot find more correct related genes, our SP-Semi-Cox is more efficient, the results proved our model has the strongest ability to find the cancer related genes.

**Figure 3:**
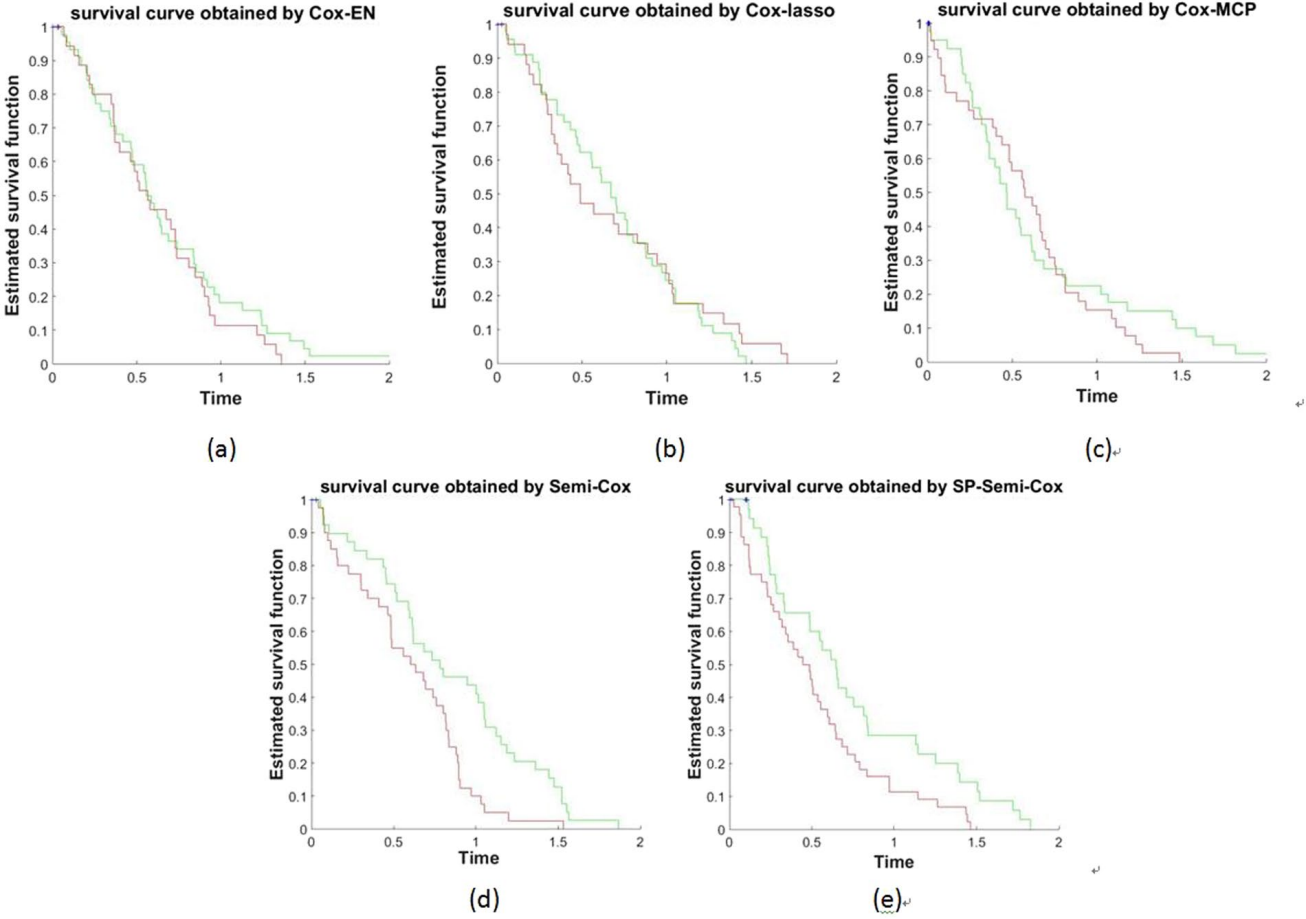
The survival curve obtained by (**a**) Cox-EN (**b**) Semi-lasso (**c**) Cox-MCP (**d**) Semi-Cox (**e**) SP-Semi-Cox.

The survival curves obtained by different Cox methods in one dataset are shown in Fig. 3, the red line is the survival curve of high risk patients, and the green line is the survival curve of low risk patients. We find the two survival curves obtained by the three supervised learning methods, both have some places overlap or intersect, however seeing the survival curves obtained by SP-Semi-Cox, the classification performance was best, and two curves with different colors did not intersect.

To further evaluate the accuracy of the model, we use the Concordance Index (CI) which can be determined as:

$$CI=\frac{{\sum }_{i}{\sum }_{j}1\,({f}_{i} < {f}_{j}\,{\rm{\& }}\,{\delta }_{i}=1)}{{\sum }_{i}{\sum }_{j}1\,({t}_{i} < {t}_{j}\,{\rm{\& }}\,{\delta }_{i}=1)}.$$

where ${t}_{i},{t}_{j}$ are the survival time of the patients *i* and *j*, *f*(.) is the survival risk function, the values of CI are between 0 and 1, and the higher value means the higher accuracy the method obtained.

**Figure 4:**
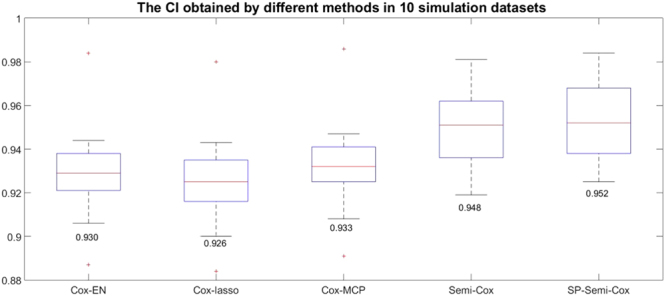
The CI obtained by different methods in 10 simulation datasets.

The average CI obtained by different methods in 10 simulation datasets are shown in Fig. 4, we can find the CI obtained by semi-supervised learning models is higher than which obtained by the three supervised learning methods, the performance of the Cox model in our semi-supervised learning model with self-paced learning is best, the Cox model in semi-supervised learning model without self-paced learning perform better than the three supervised learning methods but worse than the Cox-SP-AFT model.

Table 3 shows the MSE of estimated time obtained by five different AFT methods in different models: the elastic net penalized AFT model (AFT -EN), the lasso penalized AFT model (AFT -lasso), the AFT mode with MCP (AFT -MCP), the AFT models in semi-supervised learning models (Semi- AFT) and the AFT models in semi-supervised learning models with SPL (SP-Semi- AFT). The last row shows the average values of the MSE obtained by different methods. It is easy to find the Semi-AFT is better than the other three supervised learning AFT models, and SP-Semi-AFT model has the best performance among these five models, it means our model with SPL can predict the patients’ survival time accurately. Comparing the MSE obtained by three supervised learning AFT models, the results are much close.

**Table 3:** The MSE obtained by different methods in different simulation dataset.

Dataset	AFT -EN	AFT -lasso	AFT -MCP	Semi-AFT	SP-Semi-AFT
1	12.47	12.86	12.61	12.06	11.40
2	13.24	13.12	13.15	13.03	12.88
3	7.71	8.08	7.89	7.62	7.21
4	3.01	3.06	2.92	2.69	2.42
5	9.77	9.76	9.84	9.52	9.48
6	8.43	8.61	8.40	8.19	7.52
7	7.54	7.64	7.76	7.44	6.00
8	9.53	9.75	9.69	9.14	9.09
9	11.17	11.32	11.14	10.93	10.85
10	8.71	8.82	8.67	8.4	7.66
Average	9.15	9.30	9.20	8.90	8.44

## Discussion

In order to further evaluate the performances of different methods, these methods were applied on four gene real datasets which were collected in Gene Expression Omnibus (GEO): *GSE3141*, *GSE10141*, *GSE22210*, *GSE26389*. *GSE3141* has the information about the gene expression profile and the clinically relevant associations with disease outcomes in cancer^28^. Some data about the patients who after undergoing potentially curative treatments for hepatocellular carcinoma were recorded in *GSE10141* ^29^. *GSE22210* contains the gene expression profiling of the breast cancer patients^30^. *GSE26389* is the dataset which contain the gene information about the gastric cancer patients^31^. Some details about these different cancer datasets are given in Table 4. The first column is the number of the genes, the second is the number of the samples, and then is the number of labeled data in the dataset (remaining data are the censored data), the last second column is the number of training data, and the last column is the number of the test labeled data we used in the experiments. The experiments results were the average values of the 100 experiments on the corresponding datasets.

**Table 4:** Details of the real cancer datasets.

Dataset	genes	samples	labeled data	training	test
GSE3141	21025	111	58	91	19
GSE10141	6145	80	32	70	11
GSE22210	1452	193	65	173	21
GSE26389	4358	206	36	62	12

The numbers of selected genes are shown in Table 5, it is easy to find no matter in which dataset, the Cox-MCP always selected the least disease related genes, the SP-Semi-Cox selected more gens than Cox-MCP but less than other three methods. Comparing the remaining three methods, the Semi-Cox selected fewer genes than the Cox-EN and Cox-lasso, the Cox-EN selected most genes in the real dataset experiments. Though the SP-Semi-Cox cannot select the least genes, its accuracy is the highest as shown in the simulation experiments; this means that researchers will be most likely to identify genes associated with the disease by using our model selected genes.

**Table 5:** The number of selected genes obtained by different methods in real datasets.

Dataset	Cox-EN	Cox-lasso	Cox-MCP	Semi-Cox	SP-Semi-Cox
GSE3141	126.71	84.57	62.08	78.14	71.65
GSE10141	104.22	71.05	50.66	73.45	68.26
GSE22210	346.83	271.69	161.83	240.40	212.57
GSE26389	145.13	92.86	48.72	87.46	78.71

The average CI obtained by different methods in different real datasets is shown in Table 6. The CI obtained by Cox-lasso is always lowest; however the gap between the three supervised learning methods is small. Compared the CI obtained by three supervised learning methods, the CI obtained by the Cox models in semi-supervised learning models were higher. We also found the performance of SP-Semi-Cox is better than Semi-Cox, it means the self-paced learning can improve the semi-supervised learning model obviously.

**Table 6:** The average CI obtained by different methods in different real datasets.

Dataset	Cox-EN	Cox-lasso	Cox-MCP	Semi-Cox	SP-Semi-Cox
GSE3141	0.838	0.832	0.841	0.858	0.862
GSE10141	0.894	0.886	0.893	0.912	0.920
GSE22210	0.905	0.895	0.900	0.923	0.932
GSE26389	0.890	0.894	0.898	0.915	0.919

Table 7 gives the MSE obtained by different methods in the real datasets. We get the same conclusion as in the simulation experiments: The SP-Semi-AFT has the best performance for predicting the patients’ survival time, and the MSE obtained by Semi-AFT without SPL is lower than the other three supervised learning AFT models. Additionally, the performance of AFT –EN is better than the AFT-MCP, and the AFT-lasso has the highest MSE in the real data experiments.

**Table 7:** The MSE obtained by different methods in real datasets.

Dataset	AFT -EN	AFT -lasso	AFT -MCP	Semi-AFT	SP-Semi-AFT
GSE3141	3.12	3.40	3.21	2.85	2.64
GSE10141	18.13	18.34	18.02	16.75	15.86
GSE22210	54.22	56.17	55.33	49.88	43.61
GSE26389	20.42	21.24	20.71	17.52	15.78

The 10 top-ranked disease related genes selected by different Cox models in different real datasets were shown in Tables 8–11, the names in bold were the selected genes by different methods, and the genes with star(*) means these gene were only selected by SP-Semi-Cox method in Cox-SP-AFT model.

**Table 8:** The selected genes obtained by different methods in GSE3141.

Rank	Gene description				
	Cox -EN	Cox -lasso	Cox -MCP	Semi- Cox	SP-Semi- Cox
1	THSD1	**GLMN**	VMO1	IGDCC4	IGDCC4
2	GIPC3	LOC653513	FABP1	**HHATL**	**HHATL**
3	**GLMN**	CRYGN	**HHATL**	FABP1	**GLMN**
4	**HHATL**	LOC654433	ANGPTL7	VMO1	FABP1
5	CRYGN	GIPC3	FABP1	CDSN	SLAMF9*
6	LOC653513	THSD1	**PXN**	NPTX2	BDNFOS*
7	PDSS2	PDSS2	MOV10L1	PRSS27	DNA2
8	**PXN**	**HHATL**	C22orf43	**GLMN**	GTF2H5*
9	BTBD19	BTBD19	**GLMN**	LOC255025	**PXN**
10	CTSZ	**PXN**	KCNMB4	**PXN**	DNA2

**Table 9:** The selected genes obtained by different methods in GSE10141.

Rank	Gene description				
	Cox -EN	Cox -lasso	Cox -MCP	Semi- Cox	SP-Semi- Cox
1	**NTRK3**	**NTRK3**	EIF3S6	MMP1	SSBP1*
2	CCT6B	**MMP1**	NSMAF	NTRK3	**NTRK3**
3	**MMP1**	CCT6B	FBLN2	SDS	**M6PRBP1**
4	PSMD1	PSMD1	**MAGEC1**	**M6PRBP1**	**RPL17**
5	**M6PRBP1**	**M6PRBP1**	**RPL17**	CHD5	CADM1
6	ESRRG	ESRRG	PSMD1	TCN2	SDS
7	**RPL17**	EIF3S6	**M6PRBP1**	GTF3C1	FBLN2
8	ACSL3	F3	RPL29	ESRRG	**MMP1**
9	MAGEC1	**RPL17**	CCT6B	FBLN2	CUL2*
10	F3	DDEF2	**NTRK3**	**RPL17**	ESRRG

**Table 10:** The selected genes obtained by different methods in GSE22210.

Rank	Gene description				
	Cox -EN	Cox -lasso	Cox -MCP	Semi- Cox	SP-Semi- Cox
1	**IFNGR1**	**IFNGR1**	**VBP1**	**VBP1**	**VBP1**
2	**VBP1**	**GNMT**	**IFNGR1**	CHI3L2	BCL2A1
3	SEMA3C	**VBP1**	**GNMT**	PTCH2	HIC2
4	**GNMT**	SEMA3C	TAL1	BCL2A1	**IFNGR1**
5	HOXA11	PWCR1	CTAG1B	FGR	**GNMT**
6	ABL2	MCAM	FABP3	SEMA3C	PTCH2
7	MCAM	HOXA11	HIC2	GNMT	AFP*
8	HS3ST2	ABL2	HS3ST2	IPF1	HS3ST2
9	PWCR1	HS3ST2	CCND1	HS3ST2	FABP3
10	IRAK3	IRAK3	PI3	IFNGR1	FGF1*

**Table 11:** The selected genes obtained by different methods in GSE26389.

Rank	Gene description				
	Cox -EN	Cox -lasso	Cox -MCP	Semi- Cox	SP-Semi- Cox
1	**CREM**	**CREM**	**PMM2**	**CREM**	**CREM**
2	**SNF8**	**SNF8**	**SNF8**	**PMM2**	EIF4H*
3	**KDM5A**	**KDM5A**	TAX1BP1	MYD88	**PMM2**
4	IL18R1	IL18R1	**RAD21**	MCM7	HIST1H1A*
5	MEF2D	**PMM2**	**HAT1**	PSMD4	**SNF8**
6	**RAD21**	MEF2D	MRPS12	**KDM5A**	MEF2D
7	VRK1	**RAD21**	VRK1	**SNF8**	PSMD4
8	MRPS12	VRK1	**CREM**	**HAT1**	**KDM5A**
9	**HAT1**	FGF9	**KDM5A**	SEMA3C	**HAT1**
10	**PMM2**	**HAT1**	FGF9	**RAD21**	**RAD21**

It is very obviously that there are many genes which are selected by different methods at the same time in different datasets, such as *PXN* in *GSE3141*, *NTRK3* in *GSE10141*, *VBP1* in *GSE2210* and *KDM5A* in *GSE26389*. *PXN* encodes a cytoskeletal protein involved in actin-membrane attachment at sites of cell adhesion to the extracellular matrix, and it has been proved to be positively correlated with the clinic pathological factors of colorectal cancer^32^. *NTRK3* encodes a member of the neurotrophic tyrosine receptor kinase (*NTRK*) family, the mutations in *NTRK3* have been proved to be associated with breast carcinomas and other cancers in clinical^33^. *VBP1* plays a role in the transport of the Von Hippel-Lindau protein from the perinuclear granules to the nucleus or cytoplasm, the mutation and loss of *VBP1* may be related to the renal-cell carcinoma development^34^. The encoded protein of *KDM5A* plays a role in gene regulation through the histone code by specifically demethylation lysine 4 of histone H3, many researchers thought this gene may play a role in tumor progression^35^.

On the other hand, the Cox-SP-AFT model selected some unique genes compared other methods, *BDNFOS* in *GSE314*, *CUL2* in *GSE10141*, *AFP* in *GSE22210*, *EIF4H* in *GSE26389*. *BDNFOS* is encoded as a member of the nerve growth factor family of proteins, and it plays a role in the regulation of the stress response which was said may be related to the lung cancer^36^. The mutational of *CUL2* may play an important role in many human cancers^37^. The alpha-fetoprotein encoded by *AFP* is a major plasma protein which is often said to be associated with hepatoma or teratoma^38^. The encoded translation initiation factors of *EIF4H* can be used to stimulate the initiation of protein synthesis at the level of mRNA utilization, controlling this gene translational may make key contribution translational control in tumor promotion^39^. These genes which are mentioned in the literature demonstrated that our semi-supervised learning model can identify the real cancer related genes on the other hand.

## Conclusion

In this paper we propose a new semi-supervised learning model by combining the Cox and SP-AFT models using cancer data of high dimension and low sample size. The Cox model is used to classify the cancer patients and then the SP-AFT model can robustly predict the censored data. The embedded self-paced learning regime helps our model learn from censored data in a purely self-paced manner. To conclude, our proposed Cox-SP-AFT model can utilize more censored samples and estimate their survival time with more accuracy. Therefore, the proposed semi-supervised system is supposed to achieve higher reliability and stability. Moreover, with the aid of SPL mechanism, this model will be an efficient and versatile tool to make great contributions in cancer survival analysis.
